# A narrative account of implementation lessons learnt from the dissemination of an up-scaled state-wide child obesity management program in Australia: PEACH™ (Parenting, Eating and Activity for Child Health) Queensland

**DOI:** 10.1186/s12889-018-5237-8

**Published:** 2018-03-13

**Authors:** Debbie L. Croyden, Helen A. Vidgen, Emma Esdaile, Emely Hernandez, Anthea Magarey, Carly J. Moores, Lynne Daniels

**Affiliations:** 1 School of Exercise and Nutrition Sciences, Faculty of Health, Queensland University of Technology, Victoria Park Rd, Kelvin Grove, Brisbane, QLD 4059 Australia; 2 Nutrition and Dietetics, College of Nursing and Health Sciences, Flinders University, Adelaide, South Australia Australia

**Keywords:** Child, Overweight, Obesity, Implementation, Up-scaling, Translational research, Health system, Health service

## Abstract

**Background:**

PEACH™QLD translated the PEACH™ Program, designed to manage overweight/obesity in primary school-aged children, from efficacious RCT and small scale community trial to a larger state-wide program. This paper describes the lessons learnt when upscaling to universal health coverage.

**Methods:**

The 6-month, family-focussed program was delivered in Queensland, Australia from 2013 to 2016. Its implementation was planned by researchers who developed the program and conducted the RCT, and experienced project managers and practitioners across the health continuum. The intervention targeted parents as the agents of change and was delivered via parent-only group sessions. Concurrently, children attended fun, non-competitive activity sessions. Sessions were delivered by facilitators who received standardised training and were employed by a range of service providers. Participants were referred by health professionals or self-referred in response to extensive promotion and marketing. A pilot phase and a quality improvement framework were planned to respond to emerging challenges.

**Results:**

Implementation challenges included engagement of the health system; participant recruitment; and engagement. A total of 1513 children (1216 families) enrolled, with 1122 children (919 families) in the face-to-face program (105 groups in 50 unique venues) and 391 children (297 families) in PEACH™ Online. Self-referral generated 68% of enrolments. Unexpected, concurrent and, far-reaching public health system changes contributed to poor program uptake by the sector (only 56 [53%] groups delivered by publicly-funded health organisations) requiring substantial modification of the original implementation plan. Process evaluation during the pilot phase and an ongoing quality improvement framework informed program adaptations that included changing from fortnightly to weekly sessions aligned with school terms, revision of parent materials, modification of eligibility criteria to include healthy weight children and provision of services privately. Comparisons between pilot versus state-wide waves showed comparable prevalence of families not attending any sessions (25% vs 28%) but improved number of sessions attended (median = 5 vs 7) and completion rates (43% vs 56%).

**Conclusions:**

Translating programs developed in the research context to enable implementation at scale is complex and presents substantial challenges. Planning must ensure there is flexibility to accommodate and proactively manage the system changes that are inevitable over time.

**Trial registration:**

ACTRN12617000315314. This trial was registered retrospectively on 28 February, 2017.

## Background

Childhood overweight and obesity is a priority health issue in both rich and poor countries throughout the world [1] due to the adverse health implications that track from childhood into adult life [2]. The costs of overweight and obesity to health care systems are indicative of this dual effect [3, 4]. Efforts to address childhood obesity must include both primary prevention and management for those children who are already overweight and obese. The World Health Organization (WHO) Commission on Ending Childhood Obesity Final Report acknowledges this by identifying the *provision of family-based, multicomponent, lifestyle weight management services for children and young people who are overweight or obese* as part of universal health coverage, as one of its six key recommendations [2].

A number of research trials have demonstrated the effectiveness of lifestyle weight management programs in primary school-aged pre-adolescent children [5]. There is reasonable consensus that programs for this age group should be family-focussed and include strategies to support behaviour changes for healthy eating and physical activity [6]. These studies have informed the development of Clinical Practice Guidelines, including those for Australia [7]. Despite this, few countries have lifestyle weight management services as part of their universal child health coverage.

The Parenting, Eating and Activity for Child Health (PEACH™) Program was developed in Australia at Flinders University in the early 2000s. The PEACH™ RCT (*n* = 169) conducted in Adelaide and Sydney evaluated a 6-month healthy lifestyle program with and without an additional parenting skills component [8]. PEACH™ was one of the first large trials to evaluate outcomes of an intervention that targeted parents as the agents of change and hence did not deliver the intervention directly to children. However, children attended physical activity sessions designed to provide fun, non-competitive activities and play that ran concurrently with parent group sessions. After 6 months, there was a 10% reduction in relative weight (child body mass index and waist circumference z-scores) (*P* < 0.001) in both groups that was maintained for a further 18 months without further intervention [8]. In 2013, upscaling to a state-wide community-based program in Queensland commenced as the PEACH™ QLD Project. The project goals, as articulated in the call for tender, were to deliver the program across the state to the families of 1400 children above the healthy weight range and thereby (i) increase parenting skills and the capacity of these families to adopt healthy lifestyle behaviours in nutrition and physical activity, and (ii) contribute to the reduction of the prevalence of overweight and obesity in Queensland children. While the main contracted project deliverable was enrolment numbers, there was a tacit expectation by both funder and the project team, that the project would also explore potential models for universal delivery of childhood obesity management services. Implementation outcomes were defined by the funder and realised using a continuous quality improvement framework. This paper aims to document the implementation and dissemination of the program to inform the upscaling of programs in other jurisdictions. We describe challenges and adaptations to the implementation plan in response to participant, service and system challenges that emerged during the phased rollout, with a focus on key learnings and insights from translation and implementation at scale. The program outcomes for enrolled children and families will be reported elsewhere.

## Methods

### Context and vision

**Fig. 1:**
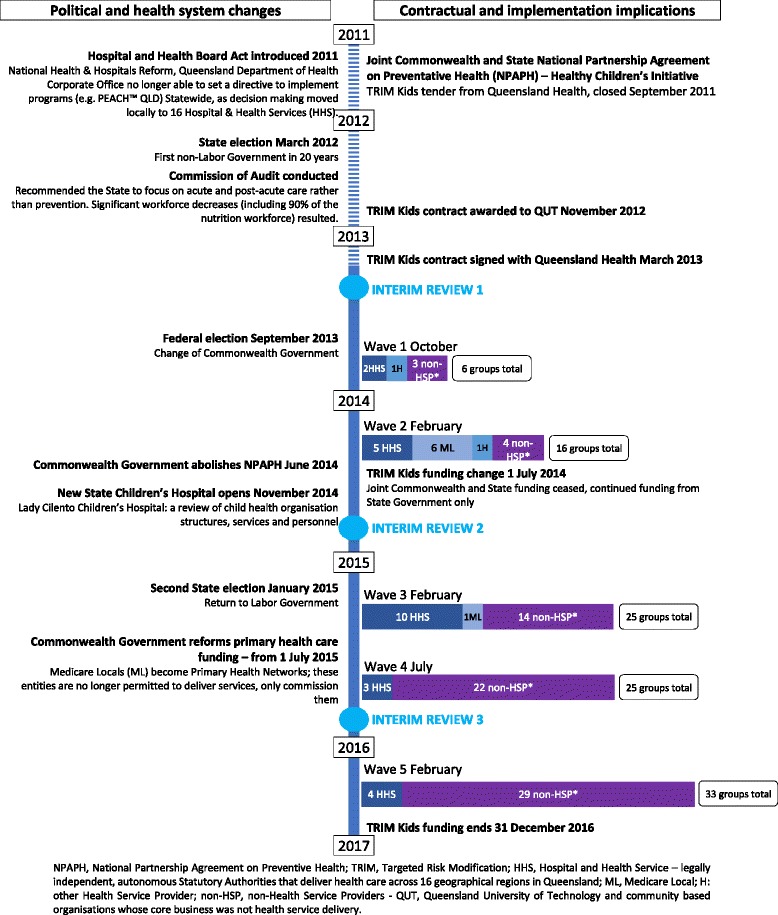
The impact of political and health system changes on the PEACH™ QLD project implementation

In Australia health care is funded both publicly and privately [9]. Responsibility for delivery of services reflects this diversity in funding. Publicly funded services may be delivered by a range of National, State and Local government service providers. Responsibility of delivery of prevention services and child health primary care services is shared. State governments typically deliver community child health services, however the National government funds general practice, which also delivers some universal child health services, using a largely private model. In response to the prevalence of childhood overweight, in 2008 the Council of Australian Governments National Partnership Agreement on Preventive Health, Healthy Children’s Initiative was established [10]. The Targeted Risk Modification Kids (TRIM Kids) Program was a Queensland government initiative under this agreement, and tenders were called for in September 2011. Queensland is the second largest state in Australia with an area of 172 7000 km^2^ with more than half its population living outside the greater metropolitan area of Brisbane [11]. The National Health Survey 2014–15 [12] prevalence data for Queensland children aged 5–17 years indicated 1 in 4 children was overweight or obese (26%) comprised of 1 in 5 overweight (19%) and 1 in 14 obese (7%). At the time of the tender call, services for overweight children in Queensland were of limited availability, fragmented, and highly variable in both quality and the extent to which they were evidence-based. In March 2013, the Queensland government, through the National Partnership Agreement on Preventive Health, funded Queensland University of Technology (QUT) to undertake this state-wide implementation of PEACH™ (referred to as PEACH™ QLD) over three years, enabled via the licencing of the program from Flinders Partners to QUT. A Project Implementation Team was established at QUT. As directed by the tender, the project outcomes were to be externally evaluated and an Evaluation Team was established at Flinders University. These health and behavioural outcomes, and reflections on the transferability of evaluation methods from a randomised controlled trial to a disseminated program, are reported elsewhere [13, 14].PEACH™ QLD’s upscaling and implementation was planned by the Implementation Team which included researchers who developed the program, along with experienced practitioners and project managers across the continuum of care. Key decision makers, advisors, practitioners and researchers with long standing experience and commitment to addressing the challenge of childhood obesity, made up the Project and Expert Advisory Committees. Advocacy for universal, accessible, evidence-based childhood obesity services delivered via the demonstration of sustainable models of care was a key goal for members of the Implementation Team and Governance Committees. The vision was that the project would work with organisations who were well positioned to deliver child obesity management services to offer them the opportunity to implement a fully funded model within their existing service. The intention was that the project would train, support and fund staff from the organisation to deliver the program under the auspices of the service. The hope was that after the project funding ceased, services would continue to deliver child obesity management services as ‘routine care’. An internal heath department survey in the first year of the project (unpublished), reported 6 of the 16 Queensland Hospital and Health Services (HHS) (independently and locally controlled statutory bodies of the Queensland government who are the principal providers of public sector health services in a geographically defined area) commit to ongoing delivery of the program following the end of the project funding period. This investment represented the first large scale state funded universal childhood overweight and obesity management program in Queensland. The project took place during a period of substantial political change that included the National Health and Hospital Reform [15], the opening of the state’s new children’s hospital and two significant changes in government at national and state level which included an audit of health services [16]. Figure 1 summarises timelines and the system and sector changes during the life of the project.

### Marketing, media and communications

From the outset the Project Implementation Team believed that strong marketing and promotional strategies were needed to raise community awareness of childhood obesity generally, promote PEACH™ QLD as an effective evidence-based response to this issue, and facilitate engagement of health services and professionals and recruitment of eligible families. A communications plan was developed and approved by the funder. A key implementation strategy was the early establishment of a dedicated marketing and communications position within the project team. Considerable investment was made to develop a range of high quality print and electronic promotional materials (including the website and social media presence) based on a recognisable design that aimed to establish the PEACH™ QLD brand. All materials were required to be approved by the funder. The wide range of marketing and promotional strategies used through the project are summarised in Table 1. An important strategy was articulation of key program messages (Table 2) that would form the basis of all media interactions. Project staff and facilitators were provided with training and laminated prompt cards to facilitate clear and consistent communication of these messages. The majority of the marketing activities were led by QUT with some site-specific promotion by local service providers and facilitators.

**Table 1:** Marketing and promotional strategies utilised by PEACH™ QLD

Marketing and promotional strategy	Examples utilised
Website presence	PEACH™ QLD website
Social media presence	PEACH™ QLD Facebook page
Media Publicity	Media releases
	Print and online articles
	Radio news reports and interviews
	Television reports
Paid advertising	Paid advertisements e.g. parenting magazines
	Paid editorials e.g. newspapers
	Commercial radio campaigns
Digital marketing campaigns	Facebook and Google Display Network
Engagement with health community, health-related organisations e.g. peak bodies and health professionals e.g. General Practitioners, Dietitians, Practice Nurses	Information disseminated through newsletters, journals, website and social media posts and dissemination of flyers.
Engagement with non-health community organisations e.g. Local Councils, Local Members of Parliament, schools, community organisations	Information disseminated through community expos, newsletters, website and social media posts, posters and dissemination of flyers.

**Table 2:** PEACH™ QLD Key Messages

Key Message
• PEACH™ stands for ‘**P**arenting, **E**ating and **A**ctivity for **C**hild **H**ealth’
• It is a free program funded by the Queensland government and delivered by QUT.
• The program promotes healthy growth in children. It is family-focussed and aims to help families lead healthier, happier lives by eating well and being more active.
• The program offers a friendly and supportive environment where parents can share their stories while children enjoy active play with new friends.
• It is evidence-based, internationally recognised and meets current guidelines and recommendations.
• To be eligible to enrol into the PEACH™ Program families must reside in Queensland and have a primary school-aged child.
• Parents/carers can register by calling the free call number or online at the program website.
• The program runs weekly within a school term and after school hours in the local community.^a^
An online version of the PEACH™ Program is also available called PEACH™ Online.^b^
• It was introduced to offer an alternative to face-to-face sessions and to ensure the program is accessible to all Queenslanders. • PEACH™ Online features 10 interactive sessions, a parent forum and facilitated ‘live-chats’. • Families can opt to do PEACH™ in one of three ways – all face-to-face sessions, all online or a combination of the two.

^a^Waves 3–5 only;^b^wave 5 only

### Program implementation plan

**Fig. 2:**
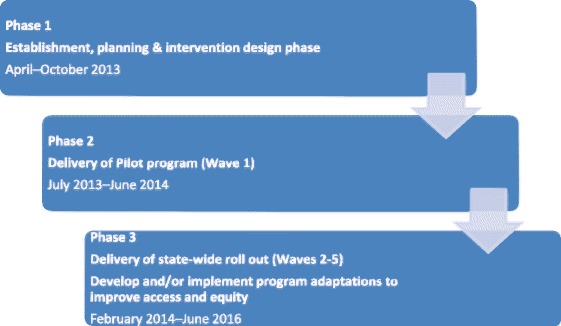
Implementation Plan timeline developed at project commencement

Program implementation took a three-phased approach as outlined in Fig. 2. The implementation plan was informed by a range of theoretical frameworks for implementation, particularly the Proctor Conceptual Model of Implementation Research [17] and the RE-AIM Framework [18]. These models also informed the type of data collected for interim program implementation reviews. The Project Implementation Team maintained a focus on service and system factors, and the Project Evaluation Team were primarily concerned with the collection and analysis of participant outcomes in response to program participation.

**Fig. 3:**
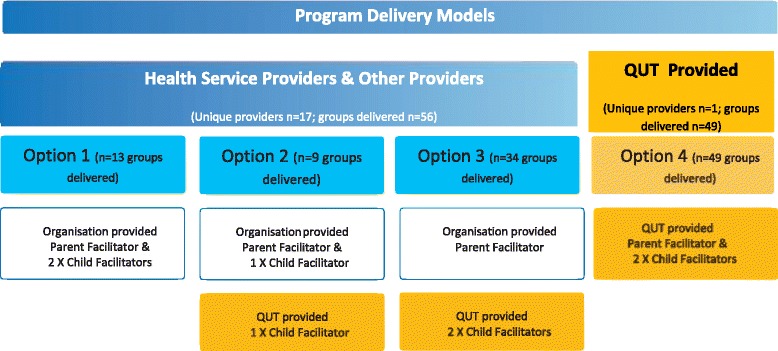
Staffing options for implementation according to the three program delivery model categories

Implementation phases 2 and 3 delivered five iterative waves based on defined time periods (essentially school terms) and geographical areas (Fig. 2). In each wave, we sought to build on the previous wave by delivering the program again at existing sites, establishing new sites and utilising both new and previously trained facilitators. To manage a range of operational issues (e.g. engagement of sites, process evaluation data availability, funding milestones) waves 1 and 2 were subsequently treated as the pilot program. We looked broadly at the type of organisations and sectors that potentially had capacity to offer the program in an ongoing service delivery model and prospectively grouped these into three provider categories for the program delivery model: (i) Health Service Providers, (ii) Other Providers, and (iii) QUT-Provided. Health Service Providers represented organisations that were publicly-funded health care providers (e.g. state-funded Hospital and Health Services (HHSs), Commonwealth-funded Medicare Locals (MLs)). Other Providers represented organisations that were from health and non-health sector agencies (e.g. allied health practices, community organisations, and universities other than QUT). We undertook extensive face-to-face advocacy with a wide range of organisations in each category and those agreeing to collaborate were funded to deliver the program via formal subcontracts. In recognition of varying capacity, services were offered three funding options based on their employee capacity to fulfil facilitator roles, as shown in Fig. 3. QUT-Provided represented a model where all aspects of program delivery (including venue sourcing) were retained by QUT, with all three facilitators employed directly by QUT.

Each wave of implementation expanded the reach and access of the program. This included geographical reach and specific strategies to reach priority population groups, such as Aboriginal and Torres Strait Islander People, those living in remote areas and culturally diverse groups. With long term program implementation in mind, the local HHS was always approached first. Where they were not interested in delivering the program, other public primary health service providers were approached and invited to express interest based on their service delivery history and access to relevant geographical areas and groups. Where there was still no interest, other non-public health organisations were invited to express interest. These included schools, sporting clubs, universities, community groups and private health practices. These organisations varied in their size, location and core business. Once a service provider had been finalised, the service and program was widely promoted to a range of other potential referrers. There was also a range of other marketing strategies to professionals including conference presentations, communication through the health department and professional bodies, and use of the networks of the professionally diverse implementation team. The Project Implementation Team collectively had substantial work experience across the health care continuum and extensive professional networks throughout Queensland. This was exploited to engage health services.

To apply a transparent and consistent funding approach to support multiple and variable sites to deliver the program, costings were modelled during the pilot phase and later refined. Health Service and Other Providers were also funded to undertake a promotion and advocacy role according to their capacity to and interest in marketing the program locally. In consultation with QUT’s legal team, a standardised subcontract was developed which was used to formalise the terms and conditions of project funding to all Health Service and Other Providers. This formal agreement carried through the terms and conditions of the Head Funding Agreement (between the Queensland Government and QUT), particularly those related to intellectual property (IP). Due to the geographical and organisational diversity of the sites and venues, occupational health and safety was a major undertaking. Site risk assessments, site inductions, careful selection of venues and documented processes ensured the programs were delivered safely.

### Recruitment and enrolment

Eligible participants as identified in the tender were children residing in Queensland, aged 7–13 years and with body mass index (BMI) 85th -95th percentiles (i.e. overweight but not obese) [19]. Based on our RCT experience and concerns regarding enrolment targets, these eligibility criteria were re-negotiated to 5–12 years (primary school-age) and BMI ≥ 85th percentile (above the healthy weight range). The aims were to (i) constrain the sample to primary school aged and thus predominantly prepubertal children, in order to limit developmental, behavioural and biological variability potentially introduced by including adolescents; (ii) maximise capacity to meet enrolment targets; and (iii) prevent potential complaints from parents of obese children seeking help but unable to participate in the program. Participants accessed the program via self- or health-professional referral operationalised via a central intake function managed by the Project Implementation Team. Families could self-refer via the website, or by toll free phone call. Health professionals referred potential participants by faxing or emailing a dedicated form directly to the central intake team. In addition, two paediatric services identified potential participant families from their waiting lists for individual dietitian appointments. Dietitians within these services used a standard protocol to contact eligible families to gain consent for their details to be provided to a project dietitian, embedded in the Project Implementation Team.

The Project Implementation Team’s project dietitian fielded the first contact calls with families to ensure the initial conversation with parents regarding their child’s weight status was positive. These calls were also used to determine eligibility based on parent-reported child height and weight, provide further information on the program, record how families heard about the program and establish the child’s suitability to participate in group-based sessions. Parents who deemed their child unable to attend group activity sessions were still strongly encouraged to enrol and bring their child to the first and last sessions to maximise the collection of pre- and post-evaluation data. Parents completed and signed consent forms, for their own and their child’s participation, at their first session.

### The intervention

PEACH™ is a group-based, 6 month family-focussed lifestyle intervention to treat overweight primary school-aged children [8, 14]. The program targets parents as the agents of change with the overall focus on improving diet quality and physical activity for the child and family. PEACH™ is guided by six overarching principles which are reflected in the content for each of the sessions: work as a family for children’s health; be role models; be balanced and consistent when parenting; base family meals and snacks on the Australian Guide to Healthy Eating; be active often and in a variety of ways; and make healthy choices easy choices [20]. The intervention initially consisted of nine × 90-min face-to-face sessions delivered fortnightly for the first 17 weeks of the 25 week program and a tenth measurement and review session at week 25. Group size was capped at 25 children per group. Parents attended sessions facilitated by a trained parent facilitator, while enrolled children participated in concurrent but separate activity sessions facilitated by two child facilitators. At these sessions children were engaged in fun, non-competitive activities and a small healthy lifestyle activity related to the content being presented in the concurrent parent sessions. The child sessions were initially planned to allow parents to attend sessions without having to arrange child care but they also served as a conduit to parents initiating a conversation with their child about healthy lifestyle change. Sessions were held outside school hours in a variety of health and community-based settings, predominantly school venues.

Parent sessions were interactive and each had defined objectives, take home messages and a small homework task. Parents were encouraged to set one or two family specific goals to work on between sessions, and encouraged to use the Planning Ahead Template (PAT) to consider and document the steps for behaviour change. Enrolled families received a 100-page, high quality printed Parent Handbook [20] and additional one-on-one parent facilitator support via three phone calls between sessions 9 and 10. Children were supported with a Child Workbook which encouraged ‘at home’ activities. Siblings were welcome to attend child sessions. Parent facilitators received two days of standardised face-to-face training or equivalent online training and were provided with a detailed program manual, material for use in group sessions and access to support from project and evaluation staff. Child facilitators, who all had experience in working with young children, underwent a two-hour induction and were provided with an induction manual and all materials and equipment to support delivery of the child sessions. A range of protocols related to child safety (including reporting responsibilities) and managing participant behaviour that was detrimental to group processes were documented and included in training. Facilitators with any concerns regarding parents or children were encouraged to contact the Project Implementation Team directly.

### Implementation review phases

During implementation, three major review phases were undertaken to address the major challenges of the project (see Fig. 1). The first review phase occurred in preparation for wave 1 pilot (mid-2013) and responded to changes in eligibility criteria. The second review phase prior to wave 3 (end-2014) occurred in preparation for delivery of the state-wide roll out and responded to the ongoing challenge of engagement of families, where attendance and retention of families was lower than expected. The final review phase occurred prior to wave 5 (end-2015) and focussed on upscaling the program for access and equity.

### Analysis

In order to analyse project implementation outcomes, and compare planned with actual implementation, project documentation and records were reviewed. During implementation, following each wave, using a cycle of continuous quality improvement, data were analysed against key performance indicators articulated by the funder. Once the project was completed, data were more strategically analysed against reach, adoption and implementation elements of the RE-AIM framework [18]. The Proctor Model was used to consider the relationship between client and implementation outcome constructs [17]. The effectiveness of PEACH™ QLD in addressing the issue of childhood obesity and maintenance of the program beyond the funding period, is reported elsewhere (paper currently under review).

Descriptive statistics obtained from participant level data were used to examine the characteristics of enrolled children and program engagement data. The sociodemographic characteristics of children enrolled in PEACH™ QLD were compared with those in the eligible population. The population eligible for PEACH™ QLD was described using data from the Child Preventive Health telephone survey 2015–16, an annual cross-sectional survey of Queensland children conducted by the Department of Health. Indicators of participant social disadvantage and accessibility were derived from residential postcode using the SEIFA Index of Relative Socio-Economic Disadvantage (IRSD) [21] and the Accessibility/Remoteness Index of Australia (ARIA2011+) [22], respectively. The four ARIA 2011+ categories range from very remote to highly accessible, and indicate the accessibility of goods and services, and opportunities for social interaction in the area. SEIFA IRSD measures are compiled by the Australian Bureau of Statistics from 2011 Census data and report relative and summary measures which represent the average of people and households in a given area. This measure summarises 17 different measures such as low income, low education, high unemployment and unskilled occupations. A low SEIFA IRSD score indicates relatively greater disadvantage in general, while a high score indicates a relative lack of disadvantage in general.

Program engagement data, including session attendance and program completion, were obtained from session sign in sheets maintained by parent facilitators. Program completers were defined as those families who attended at least 7 of the available 10 PEACH™ sessions. Data analysis was performed in SPSS 23.0 (IBM Corp) [23].

## Results

PEACH™ QLD encountered key reach, adoption and implementation challenges that were underestimated by the Project Implementation Team during initial program planning. These were categorised as (i) engagement of the health system; (ii) recruitment and promotion; and (iii) participant engagement. These challenges were considered at each review phase and responses and strategies are summarised in Table 3. Results describe client, setting, service and system factors which contributed to implementation outcomes.

**Table 3:** PEACH™ QLD implementation challenges and strategies

Implementation challenge	Strategies utilised to respond to implementation challenge			Resulting change
	Interim Review Phase 1: Responding to contractual changes and preparing for pilot.	Interim Review Phase 2: Responding to up-scaling for retention and engagement and preparing for state-wide roll out.	Interim Review Phase 3: Responding to access and equity.	
Engagement of the health system and ownership of the health issue	• Stakeholder engagement to align with key health reform policies and strategies in Qld. • Mapped workforce. • Targeted service providers versus individual health professionals. • Designed flexible program delivery model. • Developed standardised Service Agreements. • Consulted Governance Committees to develop cost modelling for program delivery. • Kept abreast of political landscape.	• Issued EOI to engage service organisations to deliver the program. • Engaged early adopters in the primary health care sector to increase diversity of health care settings. • Lobbied for the project to continue with the change in government in 2014 and demise of Commonwealth prevention funds. • Kept abreast of political landscape.	• Issued EOI to engage service organisations to deliver the program. • Advocacy work at Departmental level to endorse the program. • Mobilised strategic advocacy for the program via stakeholder presentations. • Linked with other large scale community-based programs to leverage opportunities. • Promoted program adaptations to encourage uptake from the health sector.	• Requirement of Implementation Team to be flexible and adaptable. • Increased communications with Funding Body to leverage sustainability options.
Recruitment of families and promotion	• Modified targets and milestone dates to align with implementation design. • Created PEACH™ QLD branding and marketing collateral. • Appointed dedicated marketing position. • Established enrolment processes and central intake functions to streamline enrolment. • Accessed demographic and prevalence data to map and profile Queensland to support likelihood of enrolments. • Devised geographical wave approach to implement the program state-wide. • Created database to capture program enquiries to inform future marketing.	• Deployed additional marketing strategies in regional and rural Queensland to reach the target audience and reduce stigma in small towns. • Modified marketing collateral to reflect changes in program design. • Strategically targeted rural areas to run the program to extend reach. • Monitored and adapted marketing and recruitment activities for different regions and holiday periods. • Initiated qualitative research looking at parents’ perspectives on factors influencing their decision to enrol [29]	• Trialled removal of weight criteria in four small communities. • Developed and launched PEACH™ Online to increase reach to families who could not access face-to-face groups. • Implemented online marketing strategy targeting regional and rural Queensland to promote PEACH™ Online. • Removed weight criteria. • Modified marketing collateral to reflect changes in eligibility criteria.	• Investment in marketing strategies which were driving enrolments. • Changes to eligibility criteria and enrolment milestone targets: ➢ Original eligibility criteria from 7 to 13 years to 5 to 11 years (aligning with typical ages of primary school in Qld), and from overweight only to overweight and obese from Wave 1. ➢ Removal of weight criteria and eligibility opened to all primary school-age children from Wave 5. ➢ Revised overall total enrolment target from 1400 to 1100. • Alternative models developed to launch in Wave 5, reported elsewhere.
Participant Engagement - attendance and retention	• Designed and introduced 15 min healthy lifestyle component to child sessions. • Blitzed marketing campaigns 2 months in advance of sessions commencing.	• Referred to Cochrane review [5] to compare attendance data to that of similar national programs. • Reviewed pilot attendance data. • Consultations to gain feedback on content matter, delivery format, frequency of sessions, suite of resources, order of parent sessions and general feedback. • Consulted enrolled families of eight Wave 2 groups to explore how family resources were being used and could be improved. • Reviewed program design and content to better align with families concerns and reasons for enrolment. • Reviewed facilitator training encouraged proactive approach in managing attendance. • Focussed on using community venues such as schools to reduce stigma. • Initiated value-add work on ‘why families enrol in PEACH™ QLD’ to assess motivation to enrol in the program. • Added PEACH™ QLD website functionality for facilitators to record attendance data in real time. • Reviewed child sessions for appropriateness and enhancement for adherence with current Physical Activity Guidelines [37]. • Reviewed content of 10 parent sessions and modified order.	• Initiated value-add work on ‘why enrolled families do not attend’ to further inform implementation.	• Changes to program design: ➢ Frequency: sessions 1–9 delivered weekly and within a school term (initially delivered fortnightly with no alignment to school term); ➢ Motivational content SMS messages sent to families between session 9 and 10; ➢ Three support phone calls more spaced out between sessions 9 and 10 (initially fortnightly); and ➢ Introduction of SMS reminders to enrolled families 24 h prior to session (Wave 3 only). • Parent sessions reordered. • New edition of Parent Handbook released: language simplified, paper quality lowered, images updated for cultural inclusiveness. • Value-add resources for families sourced. • Goal setting tool introduced. • Child sessions enhanced to address child satisfaction, benefit diversity in child facilitator experience and style, guide delivery of sessions for varying group sizes, levels of complexity, venues and age appropriateness.

### Engagement of the health system and ownership of the health issue

The far-reaching system changes (Fig. 1) at both state and national level required substantial adaptation of the original implementation plan. In 2011, service delivery decisions within Queensland were decentralised and devolved to newly created HHSs, which were legally independent, autonomous Statutory Authorities [24]. In addition, the Commission of Audit Report resulted in a greater emphasis on financial management, subsequent short term economic rationalism and therefore an increased focus on acute services, waiting lists and length of stay with a concomitant decreased emphasis on prevention and health promotion in HHSs [16]. An example of the impact of these changes was the almost total demise of the community nutritionist workforce [25] who in the original implementation plan had been the primary workforce target for capacity building and program delivery. The overall effects appeared to be that newly formed autonomous HHSs placed a low priority on prevention, including childhood obesity, and substantially reduced capacity to deliver healthy lifestyle promotion services. Simultaneously state-level health services for children across Queensland were being reorganised in the context of the development of the new state children’s hospital, requiring the merge of two existing childrens’ hospitals. This resulted in instability of employment for those working in child health and again non-acute services to support secondary prevention of childhood obesity were not a priority. In summary, there was little appetite within the health sector in Queensland to ‘own’ childhood obesity, despite the project funding available. Our initial response to the difficulties with engaging state level service providers was to begin advocacy with the Medicare Locals (MLs) to offer the program, directly or indirectly. The ML were entities created by the National government to geographically cluster and co-ordinate General Practices which included the ability to deliver or commission services in response to local health needs. However, by the end of the pilot phase there had been a change of national government which saw a commitment to change the governance of General Practice, most significantly, the dissolving of MLs. The ensuing uncertainties around funding and structures meant the MLs were reluctant to commit to program delivery after wave 2, see Fig. 1.

The culmination of national primary healthcare funding changes and the state move to decentralised HHSs meant a significant loss of health system engagement and capacity to deliver programs such as PEACH™. As a result, the Project Implementation Team relied much more heavily on Other Providers and QUT-Provided groups in order to meet contractual obligations (refer to Fig. 1). QUT-Provided groups made up the largest proportion overall among Service Providers (see Table 4).

**Table 4:** Summary of groups delivered, and children enrolled, by Service Provider type

Provider Type		Unique Providers *n* (%)	Groups delivered *n* (%)	Summary of enrolled families			Summary of enrolled children		
				Number enrolled *n* (%)	Number attended ≥1 session *n* (%)	Session attendance^a^ (median (IQR))	Number enrolled *n* (%)	Number attended ≥1 session *n* (%)	Session attendance^a^ (median (IQR))
Health Service Providers (HSP)	Hospital & Health Services (HHS)	5 (28)	24 (22)	252 (27)	179 (26)	6 (3–9)	294 (26)	214 (25)	6 (3–9)
	Medicare Locals (ML)	3 (17)	6 (6)	43 (5)	35 (5)	5 (2–7)	45 (4)	35 (4)	5 (2–7)
	Hospitals (H)	1 (5)	2 (2)	18 (2)	17 (2)	5 (3–9)	21 (2)	20 (3)	8 (3–9)
	*Sub-total*	*9 (50)*	*32 (30)*	313 (34)	*231 (33)*	*6 (2–9)*	*360 (32)*	*269 (32)*	*6 (2–9)*
Non-Health Service Provider (non-HSP)	Other Providers	8 (45)	23 (22)	187 (20)	146 (21)	7 (4–9)	224 (20)	178 (21)	7 (4–9)
	QUT-Provided	1 (5)	50 (48)	419 (46)	316 (46)	7 (3–9)	538 (48)	402 (47)	7 (4–9)
Total		18	105	919	693	7 (3–9)	1122	849	7 (3–9)

^a^median attendance refers to median number of sessions attended by those who joined at least one session

Of the 56 groups delivered the majority (34, 61%) undertook Options 2 and 3 of the program delivery model (refer to Fig. 3 for description of options), requiring QUT to source and employ at least one of the child facilitators. It is of note that only five of the 16 established HHSs in Queensland delivered any groups.

### Recruitment of families and promotion

Between October 2013 and September 2016, 1513 children (1216 families) enrolled in the PEACH™ Program. This comprised 1122 children (919 families) enrolled in the face-to-face program and 391 children (297 families) in PEACH™ Online. Data from PEACH™ Online families are not included due to substantial modification of the evaluation framework and format for the online version. One hundred and five (105) face-to-face groups were delivered at 50 unique venues, within 13 of 16 Queensland HHS geographic areas. However, as noted above only five of the sixteen HHS delivered the program in these areas. Of the enrolled children, 48% were boys, 30% and 51% were classified in the bottom two (greatest relative disadvantage) and top two (greatest relative advantage) SEIFA IRSD quintiles, respectively [21]; and 18% lived in moderately accessible or remote areas [22]. Seventy-eight percent of parents were born in Australia and 5% identified as Aboriginal and/or Torres Strait Islander.

**Fig. 4:**
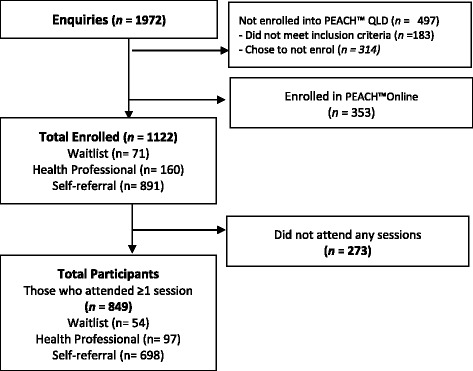
Flow chart of child participants in PEACH™ QLD Face to Face sessions

Self-referral, via the website or toll-free phone number, generated 68% of enrolments with a further 12% of families self-referring after hearing about the program from a health professional. The remaining 20% of enrolments were directly referred by a health professional (14%) or from a hospital waiting list (6%). The importance of self-referral, evident even from the pilot phase, informed the extensive marketing and promotional activities (see Table 1 and Fig. 4). For enrolments arising from self-referral, the most common methods for registration were via the PEACH™ QLD website (56%) and the toll-free number (23%). For self-referring enrolments the most successful marketing strategies were advertisements in school newsletters (20%), followed by media (14%) and print advertising (11%). Word-of-mouth contributed a further 9% of enrolments and online searches leading to the PEACH™ QLD website contributed to 6% of enrolments. The Project Implementation Team worked with the local service providers to customise marketing strategies to target priority populations in each site, for example, in regional areas local media outlets were used, notices in local stores and community notice boards and community events. At least 207 Queensland and national mainstream media reports were achieved, comprising more than 133 print and online articles, 66 radio news reports and interviews and 8 TV reports. More than 30 media releases were issued by the QUT Marketing and Media Services to raise the profile of PEACH™ QLD in the community.

### Participant engagement – Attendance and retention

Figure 4 describes the flow of participants through the program. In the pilot phase (waves 1 and 2) 250 families enrolled, of whom 25% did not attend any sessions. Of those families who attended at least one session, the median number of sessions attended was 5 and 43% completed the program. As planned, we undertook a comprehensive review at the end of the pilot phase. This included consideration of the above engagement (recruitment and attendance) outcomes, the related challenge of meeting our contractual enrolment targets, qualitative process feedback from facilitators and some parents and consultation with our Expert Advisory Committee. As a result, a number of actions and changes were implemented and are summarised in Table 3. In the subsequent waves, (waves 3–5) with 872 families enrolled, 28% of families did not attend any sessions, the median attendance was 7 sessions and 56% of families completed. Overall, the median attendance was 7 sessions and 53% of families completed.

The review also considered aspects of the program format and content resulting in changes as summarised in Table 3.

## Discussion

The translation of the PEACH™ Program from RCT to a large scale state-wide intervention presented a range of challenges, expected and unexpected, that required iterative adaptions to the original implementation plan. The Dynamic Sustainability Framework (DSF) highlights that sustained implementation requires an acknowledgement of and response to the ongoing state of change in health care systems [26]. This paper documents the impact of political change on the inter-related ecological systems, practice setting and the implementation of an intervention as described in the DSF. Our findings suggest a hierarchical relationship between these three levels. Substantial unexpected changes to the health sector undermined the planned PEACH™ QLD program delivery model, and likely added to our difficulty in engaging health care providers to deliver the program. In response, QUT became the predominant program provider rather than existing health services as was anticipated. Although this enabled the contracted enrolment targets (families and groups) to be met, this change did not support the original vision for sustainability beyond the funding period. A second major, albeit expected, challenge was enrolment and retention of families which adversely affected the capacity to meet contractual obligations regarding enrolments and to demonstrate improvements in parenting and lifestyle behaviours. Changes to program format (e.g. alignment with school terms) and content and eligibility criteria as well as increased investment in marketing and promotion enabled achievement of renegotiated enrolment targets (reduced from 1400 to 1100). While enrolment targets were met we were unable to secure meaningful health service engagement. Consequently, PEACH™ program delivery was not able to be embedded as usual care and a part of universal evidence-based childhood obesity services in Queensland.

### Engagement of the health system and ownership of the health issue

Although the need to prevent and manage childhood obesity is widely acknowledged, responsibility for providing such services in Australia remains unclear. This lack of clarity results in shifting and inconsistent ownership of the delivery of childhood obesity management services as demonstrated in the impact of political changes on the composition of service providers shown in Fig. 1. This occurs particularly at times of significant change within the system when even existing services become vulnerable. For PEACH™ QLD, the system changes meant unpredictable and variable service availability across 16 new autonomous HHSs. Translation of evidence-based programs to practice is complex due to interacting factors such as the availability of funding, organisational capacity and priorities, and time and capability constraints of practitioners [27]. Negotiations with established service providers to deliver the program via existing staff, even with initial funding available for both delivery and training, were time consuming and challenging. The temporary nature of funding was commonly raised as a barrier. Leadership, organisational culture, and appetite for embedding such a program in their existing services and strategic direction were all important to the outcome of these partnership negotiations. The autonomous statutory authority status of the HHSs meant that the funder (the Queensland Department of Health) has little capacity to directly influence the level of uptake. An important learning was that an organisational approach that engaged senior management within services prior to identifying facilitators was more successful. Generating enthusiasm in (and training) individual practitioners who then could not secure management support to run the program was inefficient and resulted in individual disappointment. As discussed above, communicating and negotiating with potential service providers was resource intensive and time and dollar costs were underestimated in the implementation timelines and budget. Few non-health service providers undertook delivery of the program. This may highlight the importance of alignment with an organisation’s core business in the viability of sustained delivery. University-government collaborations are common during program trials. These collaborations add rigour to findings and potentially influence future practitioners, however they are not a long term solution to universal health service delivery.

The original tender was conceived in part, in response to clinicians’ concern regarding waiting lists and purported demand for a universal service. Our preferred program delivery model assumed publicly-funded health service providers were well positioned and resourced to deliver the program and ultimately embed it within their usual service. There are well documented examples [28, 29] where childhood obesity management programs have been integrated into publicly-funded health services e.g. Local Health Districts, the New South Wales equivalent to HHSs in Queensland. Despite investigating HHSs (*n* = 16) interest in sustained program implementation beyond the project funding period before the implementation trial began, only 5 accepted project funding to directly deliver the program and none delivered groups across all waves. In response, QUT either delivered or subcontracted private healthcare providers to deliver the program thus limiting sustainability beyond the funding term and raising important questions about the role of a university as a service provider.

### Implementation at scale

For projects delivered at scale, a substantial establishment phase with appropriate funding and milestones is required to enable development of effective governance, stakeholder partnerships, costing models, communication strategies, referral pathways, protocols and ethics approvals and workforce recruitment and training before program delivery commences [30]. The six-month period between contract finalisation, hence access to funding, and commencement of the first groups (a milestone with funding attached), was inadequate. This was exacerbated by the 18-month period between submission of response to the tender (i.e. development of the initial implementation plan) and the award of the tender to QUT. A major learning was that an appropriate length establishment phase must be planned and funded in the context of the expected timelines for commencement of program delivery.

PEACH™ QLD applied a horizontal approach [31] to scaling up, introducing the PEACH™ intervention across different sites via the geographical wave approach. In our experience, there were a number of benefits to this approach e.g. cost and resource efficiencies in scheduling pre-wave facilitator training. Operational efficiencies were found in offering group sessions weekly to align with school terms, particularly school terms 2 and 3, in metro/inner regional areas, more than once in an area and via the QUT-Provider model. It appears that this modification, implemented for waves 3–5, also improved attendance and completions. The saturation point (i.e. maximum number and frequency of viable groups per geographic location) could not be determined within the short-term project funding period but is an important implementation metric in considering sustainability at a particular site.

### Recruitment of families and promotion of the program

A key learning was the importance of self-referral and the positive role that schools can play in publicising the program. A disappointingly low proportion of referral from health professionals reinforced our decision to invest heavily in high quality professional marketing of the program. This included development of a suite of high quality, consistently branded promotional materials to engage health professionals, organisations and the community. It remained an ongoing challenge to ensure media stories focussed on positive messages and imagery and avoided blaming and negative stereotyping families, hence it was important to carefully vet all media releases. Where possible, experienced senior project staff handled radio and television interviews. Media training and support of project staff, including facilitators endeavoured to ensure PEACH™ QLD messages were communicated consistently, positively and effectively at the local level. Marketing efforts focussed on a program empowering parents to seek help and support to improve child and family health. It is possible that the campaign had an impact beyond enrolled families in terms of raising community awareness of childhood obesity. While the project did not target eligible children in promotional materials, anecdotal reports from facilitators suggested children influenced family attendance so should be considered in future marketing activities. The interaction between parent and child in the family response to improve child weight status requires further exploration. Any marketing strategies to directly target children will need careful planning to avoid unintended adverse consequences for the children and their families.

Our experience suggested recruitment of families was effective when centralised i.e. eligibility, program information and enrolment was managed by the project dietitian as the first point of contact with families; commencement dates, times and locations were confirmed prior to marketing and specified in local publicity; and time between promotion, enrolment and starting groups was minimised, with < 6–8 weeks being optimal. Effective marketing and promotion were critical as the majority of enrolled families self-referred, with promotion through schools being the most effective marketing strategy. This finding is consistent with a study by Welsby et al. [29]. Health professional response and referral was disappointing and conversions from established hospital waiting lists were particularly poor. This is consistent with the overall difficulty in engaging publicly-funded health providers and highlights that ongoing service availability and routinely monitoring child weight status resulting in program referral are inter-related.. Activity based funding of services structured around individual appointments and parent expectations related to this more traditional model of care may also have been barriers. Furthermore, it is possible that health professionals who had little experience with group-based obesity management programs, or were not aware such programs are encouraged by the national clinical practice guidelines for obesity management, did not ‘sell’ the program well to families.

Despite endeavouring to run the program in small rural communities throughout Queensland, it was often not possible to secure sufficient enrolments to ensure groups were viable. Further, in larger centres, it was also difficult to achieve viable enrolments where programs were run in two consecutive school terms, indicating that careful planning of site locations and adequate spacing of programs across school terms is important. Despite intensive promotional activities our enrolment numbers did not always meet minimum numbers required for group viability in terms of both fixed costs of delivery and group function. In some instances, groups were cancelled or delayed to future terms due to low enrolment numbers. This resulted in disappointment for families who had enrolled and facilitators who were expecting to consolidate their training and run groups. The challenge of enrolment is likely to reflect the well-documented complexity of parental awareness of and readiness to change their child’s weight [32] rather than the lack of eligible families within the community. Anecdotally, there appeared to be an increase in program enquiries and enrolments where the program was conducted more than once, possibly due to greater awareness in the community and positive word-of-mouth publicity. This is consistent with the experience of the NSW Go4Fun program [29], and suggests that repeated delivery at a consistent site assists with establishing the program within the community. In addition, there were operational efficiencies associated with repeating programs such as availability of trained local facilitators, established venues with completed health and safety assessments and hire agreements and child session equipment.

Reach is a fundamental aspect of implementation at scale [33]. Despite the challenges, recruited families were reasonably representative of eligible families (child 5–11 years of age and above the healthy weight range) across Queensland [34]. Overall, we were able to reach families with relative disadvantage and living in less accessible areas of Queensland.

### Participant engagement (attendance and retention) of families

Successful outcomes of childhood weight management programs rely on family engagement [35]. This was a key challenge. One in four families who completed the enrolment process did not attend any sessions. These families generally did not complete baseline assessment and hence, there are little data available to assist in understanding the reasons behind non-attendance. Benchmarking PEACH™ QLD against other large scale childhood obesity management programs is difficult as engagement, attendance and completion are inconsistently defined, and in particular, the proportion of enrolees never attending is rarely reported in the literature. However, other large scale paediatric healthy lifestyle interventions [36–38] report completion rates (defined between ≥ 70–75% of available sessions) of 45–54%, comparable to PEACH™ QLD.

Engagement reflects complex interactions at the family level between prior experience, current perceived needs, readiness for change, influence of significant others and feasibility of attending sessions. We considered attendance in PEACH™ QLD a crude surrogate measure of this complexity. The role of other group members, group size and dynamics and facilitator experience, confidence and enthusiasm are also potential factors that may influence attendance. In PEACH™ QLD, the role of facilitators on attendance was underexplored but anecdotally, both parent and child facilitator capability and enthusiasm had an impact on retention. A systematic review by Kelleher et al. [35] investigated factors influencing attendance at community-based lifestyle programs of families of overweight or obese children. The review concluded that whilst parents provided the impetus for program enrolment, children’s positive social experiences, such as having fun and making friends, fostered the desire to continue. The PEACH™ QLD intervention targeted parents not children. As in the RCT [8], the child sessions were offered primarily as child minding to facilitate parent attendance. However, considerable effort was invested to make these sessions relevant and fun. Our experience from both the RCT and the upscaled program suggests the sessions were enjoyed by the children and encouraged attendance, consistent with the conclusion of the Kelleher review [35]. As suggested by Kelleher et al. [35], it may be important for recruitment strategies to target parents and retention strategies to focus on both parents and children.

Several modifications may have enhanced participant engagement beyond pilot phase including changes in format e.g. frequency of sessions from fortnightly to weekly and timing within the school term; shift from healthcare to community-based venue settings; enhancements to program content and materials. As noted above, an inherent challenge is that parents who do not attend any sessions; withdraw formally or otherwise stop attending, do not complete baseline and/or outcome assessment. Hence, there are limited data to enable examination of reasons for poor engagement or the impact of lower attendance (a proxy for intervention dose) on improvements in healthy lifestyle indicators. Parents may stop coming due to a range of factors related to satisfaction and/or logistics. It is also plausible that at some point they feel that the program has met their perceived needs for information and support and they do not need to continue to attend. Characterising how and why families ‘engage’ and meet their perceived needs is complex and to date has received limited attention. Planning, particularly in terms of ethics approval and resource allocation, is required to enable follow up of families who do not attend, to inform development of retention strategies. A practical aspect of poor engagement (never attending/attrition) is the impact on viable group size both in terms of the fixed costs of offering the group at all and group dynamics if the number of families regularly attending drops too low. The initial implementation plan worked on enrolling 15 children per group. This target was rarely met which had a range of resource and operational implications.

Balancing strategies to maximise attendance with best practice in childhood obesity management is complex. The PEACH™ Program is consistent with clinical practice guidelines [7] and evidence reviews [5] which recommend a longer period of family engagement e.g. 6 months, in order to facilitate sustainable behaviour change. However, the majority of enrolled families did not attend the final session at 25 weeks. While it is tempting to reactively and iteratively modify programs in an attempt to align with the needs of families (identified to variable extent via process evaluation), this must be balanced against the need to implement evidence-based approaches. Within a quality improvement framework we modified a number of program elements on the basis of interim evaluation data, informal feedback, and review of newly published literature to adapt to both operational and participant needs. However there is inherent tension between responding to process evaluation when up-scaling a program and maintaining the integrity of the original evidence-based intervention. There are also tensions in the requirements of an evaluation framework and associated ethics approval (including for all iterative variations to the program and data collection tools) designed to evaluate implementation and outcomes of an up-scaled program conceptualised as service provision versus translational research [13]. Further consideration is required regarding the scope and process of ethics approval of translational research projects that seek to evaluate up-scaling of interventions already demonstrated to be efficacious that are effectively being implemented in a service provision framework.

## Conclusions

PEACH™ QLD successfully up-scaled the evidence-based PEACH™ Program by reaching over 1200 families and over 1500 children across Queensland. The cohort was broadly representative of Queensland families with primary school-aged children above the healthy weight range. Contractual enrolment targets were met but recruitment and engagement of families were challenging, although consistent with similar programs. Key learnings for the dissemination of a program at scale include (i) the need to plan and fund adequate lead times to set up governance and operational procedures prior to commencement of program delivery; (ii) the importance of high quality marketing and promotion to support self-referral to the program; (iii) the operational efficiencies of aligning with school terms and engaging schools in promotion; and (iv) the need to ensure process evaluation is structured to provide detailed real-time information on the needs and responses of families, regardless of level of engagement, to inform program modifications within a continuous quality improvement framework. Despite the prevalence of childhood obesity worldwide [1], and the large number of programs that have been developed to address it [5], this paper is one of the few to document the experience of real world implementation of childhood obesity management services at scale. Recommendations for dissemination of an upscaled program are described in Table 5.

**Table 5:** Recommendations for the dissemination of an upscaled child obesity management program

1. Health system	Recommendation 1.1: Governments should continue to invest in community based healthy lifestyle programs for families as part of a universal service delivery model for the treatment of overweight and obese primary school aged children. This investment should be embedded in the policy and strategic context for community and primary care. Recommendation 1.4: Primary health care services are well positioned for ongoing service provision of childhood overweight and obesity management services. Recommendation 1.2: Government investment in programs should include routine performance monitoring for participating health services and program monitoring to ensure health gains continue to be achieved. Programs should include a quality improvement cycle to improve outcomes. Recommendation 1.3: The establishment and monitoring of local level performance targets for program delivery are needed to ensure childhood obesity management services are prioritised by providers.
2. Implementation at scale	Recommendation 2.1: A Model of Care and implementation plan should be closely aligned with and informed by national Clinical Practice Guidelines and the WHO Commission on Ending Childhood Obesity Report. Recommendation 2.2: A consistent and coordinated approach to paediatric weight management services across the State including eligibility, availability and type of service with the ability to deliver services to families at the point in which people engage with the health care system. Clinical Practice Guidelines should be extended to describe care pathways with the approach involving all National, State and Private health service providers involved in the management of childhood overweight and obesity across the continuum of care in clinical, community and primary health care settings across a complexity of cases. A shared vision by all, referral pathways and communication between providers is needed, as services to families of children who are already overweight or obese do not sit in only one part of the health care continuum. Recommendation 2.3: Upscaling of programs requires a deep understanding of administrative enablers and barriers to embedding childhood obesity management into the core business of the health service, including links to internal funding structures and medical record charting of occasions of service.
3. Recruitment of families and promotion of the program	Recommendation 3.1: Programs should market directly to families as it is unlikely that the families who would benefit from the program are all interacting with the health care system. Health professional referrals alone are not adequate. Recommendation 3.2: Children impacted on parent’s interest in taking action on their weight status. Marketing directly to children is likely to be complex but warrants further investigation. Recommendation 3.3: Care should be taken to not further sensationalise childhood obesity in the media, and therefore it is critical to ensure that marketing is solution focussed and not problem focussed.
4. Engagement of families	Recommendation 4.1: Future investment and expansion of PEACH™ must apply a quality improvement framework to ensure modifications and changes which led to an improvement can conclusively demonstrate they are worthwhile. Recommendation 4.2: The evaluation of up-scaled programs should focus on reducing the burden on participant families whilst maintaining the integrity of evaluation datasets for ongoing monitoring and surveillance.

The most difficult challenge was engaging the health care sector and ensuring sustainability of the program beyond the funding period. Childhood obesity management is a complex practice and service provision problem. Since completion of the funded project, PEACH™ QLD, is no longer available to Queensland families. PEACH™QLD aligned with multiple government priorities and policies which strongly articulate the need to address childhood overweight and obesity and increase the capacity of health professionals and services within the health sector to do so [39–42]. Despite this, the provision of “appropriate weight management services for children and adolescents who are overweight or obese that are family-based, multicomponent (including nutrition, physical activity and psychosocial support) and delivered by multiprofessional teams with appropriate training and resources, as part of universal health coverage”, as recommended by the WHO Commission on Ending Childhood Obesity [2], is yet to be achieved. The experience of PEACH™QLD reflects the broader question of where responsibility lies for provision of universal services to prevent and manage childhood obesity and what ecological system and contextual setting factors may be required to realise this global target.

## Abbreviations

ARIA: Accessibility-remoteness index of Australia
BMI: Body Mass Index
GP: General Practitioner
HHS: Hospital and Health Service
IRSD: Index of Relative Socio-Economic Disadvantage
LHD: Local Health Districts
NPAPH: National Partnership Agreement on Preventive Health
PAT: Planning Ahead Template
PEACH™: Parenting, Eating and Activity for Child Health
QLD: Queensland
QUT: Queensland University of Technology
SEIFA: Australian Bureau of Statistics’ Socio-Economic Indexes for Areas
TRIM Kids: Targeted Risk Modification Kids
